# Functional variants of the pentraxin 3 gene are associated with the metastasis and progression of prostate cancer

**DOI:** 10.1111/jcmm.70041

**Published:** 2024-08-26

**Authors:** Wei‐Chun Weng, Yi‐Hsien Hsieh, Chia‐Yen Lin, Yu‐Fan Liu, Shih‐Chi Su, Shian‐Shiang Wang, Shun‐Fa Yang

**Affiliations:** ^1^ Division of Urology, Department of Surgery Tungs' Taichung Metroharbor Hospital Taichung Taiwan; ^2^ Department of Post‐Baccalaureate Medicine, College of Medicine National Chung Hsing University Taichung Taiwan; ^3^ Institute of Medicine Chung Shan Medical University Taichung Taiwan; ^4^ Department of Medical Research Chung Shan Medical University Hospital Taichung Taiwan; ^5^ Division of Urology, Department of Surgery Taichung Veterans General Hospital Taichung Taiwan; ^6^ School of Medicine Chung Shan Medical University Taichung Taiwan; ^7^ School of Medicine National Yang Ming Chiao Tung University Taipei Taiwan; ^8^ Department of Biomedical Sciences Chung Shan Medical University Taichung Taiwan; ^9^ Whole‐Genome Research Core Laboratory of Human Diseases Chang Gung Memorial Hospital Keelung Taiwan; ^10^ Department of Medical Biotechnology and Laboratory Science, College of Medicine Chang Gung University Taoyuan Taiwan; ^11^ Department of Applied Chemistry National Chi Nan University Nantou Taiwan

**Keywords:** metastasis, pentraxin 3, prostate cancer, single‐nucleotide polymorphism

## Abstract

Age, ethnic background and genetic components have been identified as the established risks for prostate cancer (PCa). Pentraxin 3 (PTX3), originally identified as a pattern‐recognition molecule for defence against infectious agents, has multiple functions in tissue repair and in the regulation of cancer‐associated inflammation. In this study, we sought to investigate the impact of *PTX3* gene variants on the development of PCa. Genotypes of four common single‐nucleotide polymorphisms (SNPs) of *PTX3* gene, including rs1840680, rs2305619, rs3816527 and rs2120243, were profiled among 705 PCa patients and 705 ethnicity‐matched controls. In this study, we found that patients who carry at least one minor allele (C) of rs3816527 (AC and CC) tended to develop advanced forms of diseases (clinical large T stage, OR, 1.593, *p* = 0.032; pathologically‐confirmed nodal spread, OR, 1.987, *p* = 0.011; metastatic tumour, OR, 3.896, *p* = 0.032) as compared with those homologous for the major allele (AA). Further stratification analysis showed that such association of rs3816527 with lymphatic and distal metastasis of PCa was accentuated in the younger age group (≤65 at diagnosis) but not seen in the older age group (>65 at diagnosis), suggesting an age‐specific effect of *PTX3* variants. Prediction of PTX3 protein structure implied that polymorphism may alter the quaternary organization and oligomerization of PTX3 protein. Moreover, our gene silencing experiments and survey of public datasets revealed that elevation of PTX3 levels in PCa was required for cell migration and associated with tumour metastasis. Our results highlight an association of *PTX3* rs3816527 with the progression of PCa.

## 1 INTRODUCTION

Prostate cancer (PCa) is the second most frequent malignancy among men globally, accounting for a substantial portion of cancer‐related deaths.^1^ Age, ethnicity and genetic factors have been identified as established risks for PCa.^2^ Study of age‐specific incidence curves revealed that the risk for PCa begins to rise sharply after the age of 55 and peaks at the age from 70 to 74.^3^ The prevalence of PCa varies greatly across different ethnic populations and geographic regions, with the highest rate among men of African ancestry and the lowest rate among Asian males.^4^, ^5^ As the presence of a familial aggregation of PCa has been proposed many decades ago,^6^ recent advancements in DNA sequencing have uncovered a number of PCa susceptibility genes that control DNA repair mechanisms.^7^ These genetic components together with numerous common germline variants that confer low to moderate risks were estimated to underpin more than a third of familial PCa risk.^8^ In addition to forementioned risks, other modifiable etiological parameters of PCa, although still debatable, include but not limited to metabolic syndrome, obesity and smoking.^2^ Taking such heterogeneous nature of disease aetiology into consideration, all potential causes stated above appear to be interconnected and needed to evaluate the occurrence and prognosis of PCa.

Pentraxins, a cyclic multimeric protein family belonging to soluble pattern recognition receptors (PRRs), are central to complement activation and innate immunity.^9^, ^10^, ^11^ Among the members of this evolutionarily conserved protein family, pentraxin 3 (PTX3) was implicated as essential components of inflammation and tissue remodelling, in addition to its fundamental roles in defence against infectious agents.^12^, ^13^ Paradoxically, PTX3 can exhibit both suppressive and promotive effects on the progression of cancer.^14^, ^15^ In line with the observations that silencing of PTX3 expression via an epigenetic manner was detected in some cancer types,^16^, ^17^, ^18^ a tumour suppressor function of PTX3 was demonstrated in gene knockout mice.^18^ However, several in vitro experiments performed with manipulation of PTX3 expression indicated that PTX3 acts as a cancer promotor through inducing epithelial‐mesenchymal transition and macrophage chemotaxis.^19^, ^20^, ^21^ These contradictory findings imply that PTX3 may play a dual role in tumorigenesis, possibly depending on the types of malignancies, or on the cells producing it within the tumour microenvironment.

Single‐nucleotide polymorphisms (SNPs) in the *PTX3* gene have been associated with the susceptibility to pulmonary aspergillosis,^22^ COVID‐19 severity,^23^ hepatocellular carcinoma,^24^ oral cancer^25^ and cervical cancer.^26^ Although extensive genome‐wide association studies have reported numerous common SNPs of modest effects on PCa risks,^27^, ^28^, ^29^, ^30^ the genetic mechanism behind such susceptibility remains still largely elusive. Here, we used a targeted gene approach with a case–control setting to assess the influence of *PTX3* SNPs on the risk of PCa.

## 2 MATERIALS AND METHODS

### 2.1 Subjects

A cohort comprising 705 PCa cases who received a robot‐assisted laparoscopic radical prostatectomy from 2012 to 2018 was recruited at Taichung Veteran General Hospital (Taichung, Taiwan) with the approval by the Institutional Review Board (CE19062A). Additionally, 705 non‐cancer males of the same ethnicity who reside in a similar geographic region were randomly selected as a control group. Informed consent and peripheral blood were collected from each subject. Demographic and clinical parameters of PCa cases at diagnosis, such as age, tumour staging, prostate‐specific antigen (PSA) levels, pathologic Gleason scoring, D'Amico risk classification and cancer invasion status (perineural, seminal vesicle and lymphovascular), were retrieved from their medical records.

### 2.2 Genotyping

Genotypes of four common SNPs in *PTX3* gene (rs1840680, rs2305619, rs3816527 and rs2120243) chosen based on their putative associations with many pathogenic conditions^22^, ^23^, ^24^, ^25^, ^26^ were determined in this investigation. The QIAamp DNA Blood Mini kit (Qiagen, Valencia, CA, USA) was used to extract genomic DNA from whole blood samples and PCa cell lines. Discrimination of alleles for four SNPs was performed via the TaqMan assay with an ABI StepOne™ Real‐Time PCR System (Applied Biosystems, Foster City, CA, USA), and then analysed by SDS version 3.0 software (Applied Biosystems, Foster City, CA, USA).

### 2.3 Prediction of PTX3 protein structure

Structural information of PTX3 protein was predicted by AlphaFold, a machine learning approach that incorporates physical and biological knowledge regarding protein structures,^31^ as the crystal structure of PTX3 has not been determined yet. Full‐length amino acid sequence of human PTX3, consisting of 381 residues, was downloaded from Uniprot (UniProt ID: P26022). Structures of PTX3 were accessed from the AlphaFold Protein Structure Database (https://alphafold.ebi.ac.uk/).

### 2.4 Immunoblotting

Protein lysates were prepared from cell culture and separated using SDS‐polyacrylamide gels, followed by transferring to Immobilon PVDF membranes (Millipore). Specific immunoglobulins targeting the following molecules were used for detection: Anti‐PTX3 and anti‐β‐actin from Abcam (Waltham, MA, USA); HRP‐conjugated secondary antibodies. Densitometry of immunoblots was carried out via ImageJ.

### 2.5 Cell culture and migration

PCa cell lines (Du‐145, PC‐3 and 22Rv1) were obtained from the American Type Culture Collection (ATCC; Rockville, MD, USA) and maintained in RPMI 1640 medium with L‐glutamine (Gibco) at 37°C in a humidified atmosphere with 5% CO_2_. The potential of PC‐3 cells to migrate was evaluated with a modified Boyden chamber experiment as described previously.^32^ Briefly, cells with/without manipulation of PTX3 expression were seeded on the 8‐μm‐pore size polycarbonate membrane filter with serum‐free media at a density of 10^4^ cells per well, allowed to migrate for 24 h, and subsequently counted under an Olympus microscope (Olympus, Tokyo, Japan).

### 2.6 Statistical analysis

Associations of *PTX3* genotypic frequencies with the occurrence or clinical parameters of PCa were assessed by logistic regression models. The difference of PTX3 expression in the datasets of the Gene Expression Omnibus (*GEO*) repository was analysed by Student's t‐test. Data were calculated by using SAS software. The threshold of difference/association was set by a *p*‐value of <0.05.

## 3 RESULTS

### 3.1 Study cohort

In this investigation, 705 male patients were recruited to explore the potential correlation of *PTX3* gene polymorphisms with the development of PCa. Analyses of demographic and clinical features demonstrated that their age tended to be high (57.7% over 65 years old), and the majority were diagnosed with low/intermediate‐graded and early‐staged tumours (Table 1). A small proportion of patients developed nodal spread (2%) and distal metastasis (1.6%). Among our cases, seminal vesicle, perineural and lymphovascular invasion occurred in 21.4%, 73.6% and 15.9% of patients, respectively. Based on the D'Amico risk scores, a total of 50.4% of patients were predicted at a high risk for disease recurrence.

**TABLE 1:** The distributions of demographical characteristics in 705 patients with prostate cancer.

Variable	Patients (*N* = 705)
Age at diagnosis (years)	
≤65	298 (42.3%)
>65	407 (57.7%)
PSA at diagnosis (ng/mL)	
≤10	335 (47.5%)
>10	370 (52.5%)
Pathologic Gleason grade group	
1 + 2 + 3	585 (83.0%)
4 + 5	120 (17.0%)
Clinical T stage	
1 + 2	607 (86.1%)
3 + 4	98 (13.9%)
Clinical N stage	
N0	691 (98.0%)
N1	14 (2.0%)
Clinical M stage	
No	694 (98.4%)
Yes	11 (1.6%)
Pathologic T stage	
2	373 (52.9%)
3 + 4	332 (47.1%)
Pathologic N stage	
N0	645 (91.5%)
N1	60 (8.5%)
Seminal vesicle invasion	
No	554 (78.6%)
Yes	151 (21.4%)
Perineural invasion	
No	186 (26.4%)
Yes	519 (73.6%)
Lymphovascular invasion	
No	593 (84.1%)
Yes	112 (15.9%)
D'Amico classification	
Low risk/intermediate risk	350 (49.6%)
High risk	355 (50.4%)
Biochemical recurrence	
No	481 (68.2%)
Yes	224 (31.8%)

### 3.2 Association of PTX3 SNP with the progression of PCa

To assess the potential link of *PTX3* gene variants to the progression of PCa, four *PTX3* SNPs (rs1840680, rs2305619, rs3816527 and rs2120243) exhibiting no significant deviation (*p* > 0.05) from Hardy–Weinberg equilibrium in controls were explored in this investigation. Although none of these SNPs reached the threshold for significant associations, marginal effects on PCa risks were observed for specific genotypes of rs1840680, rs2305619, rs3816527 and rs2120243 after the adjustment for age (Table 2). Moreover, we tested the impact of *PTX3* polymorphism on clinicopathological parameters of PCa patients (Tables 3 and 4). We found that patients who bear at least one minor allele (C) of rs3816527 (AC and CC) tended to develop advanced forms of diseases (clinical T stage III/IV, OR, 1.593; 95% CI, 1.038–2.444; *p* = 0.032) (pathologically‐confirmed nodal spread, OR, 1.987; 95% CI, 1.164–3.391; *p* = 0.011) (metastatic tumour, OR, 3.896; 95% CI, 1.025–14.812; *p* = 0.032) as compared with those homologous for the major allele (AA) (Table 3). Yet, such association with advanced PCa was not detected in rs1840680, rs2305619 and rs2120243 (Tables 3 and 4). These results highlight a possible connection of rs3816527 variants with the progression of PCa.

**TABLE 2:** Adjusted odds ratio (AOR) and 95% confidence interval (CI) of prostate cancer associated with *PTX3* genotypic frequencies.

Variable	Controls (*N* = 705) (%)	Patients (*N* = 705) (%)	AOR (95% CI)	*p*‐value
rs2120243				
CC	308 (43.7%)	297 (42.1%)	1.000 (reference)	
CA	329 (46.7%)	328 (46.5%)	1.065 (0.818–1.385)	0.641
AA	68 (9.6%)	80 (11.4%)	1.185 (0.777–1.807)	0.431
CA + AA	397 (56.3%)	408 (57.9%)	1.086 (0.844–1.397)	0.524
rs3816527				
AA	437 (62.0%)	415 (58.9%)	1.000 (reference)	
AC	234 (33.2%)	258 (36.6%)	1.272 (0.978–1.655)	0.073
CC	34 (4.8%)	32 (4.5%)	0.874 (0.479–1.595)	0.661
AC + CC	268 (38.0%)	290 (41.1%)	1.217 (0.945–1.567)	0.128
rs2305619				
GG	295 (41.8%)	274 (38.9%)	1.000 (reference)	
GA	328 (46.5%)	347 (49.2%)	1.291 (0.988–1.685)	0.061
AA	82 (11.7%)	84 (11.9%)	1.228 (0.815–1.850)	0.327
GA + AA	410 (58.2%)	431 (61.1%)	1.278 (0.990–1.647)	0.059
rs1840680				
GG	316 (44.8%)	295 (41.8%)	1.000 (reference)	
GA	309 (43.8%)	330 (46.8%)	1.298 (0.976–1.731)	0.063
AA	80 (11.4%)	80 (11.4%)	1.132 (0.748–1.712)	0.557
GA + AA	389 (55.2%)	410 (58.2%)	1.286 (0.999–1.656)	0.051

*Note*: AOR with their 95% confidence intervals were estimated by multiple logistic regression models after controlling for age.

Abbreviation: AOR: adjusted odds ratio.

**TABLE 3:** Odds ratios (ORs) and 95% confidence intervals (CIs) of the clinical status and *PTX3* rs2120243 and rs3816527 genotypic frequencies in 705 patients with prostate cancer.

Variable	rs2120243				rs3816527			
	CC (*N* = 297)	CA + AA (*N* = 408)	OR (95% CI)	*p*‐value	AA (*N* = 415)	AC + CC (*N* = 290)	OR (95% CI)	*p*‐value
PSA at diagnosis (ng/mL)								
≤10	142 (47.8%)	193 (47.3%)	1.000	0.894	206 (49.6%)	129 (44.5%)	1.000	0.177
>10	155 (52.2%)	215 (52.7%)	1.021 (0.757–1.377)		209 (50.4%)	161 (55.5%)	1.230 (0.910–1.662)	
Pathologic Gleason grade group								
1 + 2 + 3	249 (83.8%)	336 (82.4%)	1.000	0.604	349 (84.1%)	236 (81.4%)	1.000	0.345
4 + 5	48 (16.2%)	72 (17.6%)	1.112 (0.745–1.659)		66 (15.9%)	54 (18.6%)	1.210 (0.814–1.797)	
Clinical T stage								
1 + 2	257 (86.5%)	350 (85.8%)	1.000	0.777	367 (88.4%)	240 (82.8%)	1.000	**0.032** *
3 + 4	40 (13.5%)	58 (14.2%)	1.065 (0.690–1.643)		48 (11.6%)	50 (17.2%)	**1.593 (1.038–2.444)**	
Clinical N stage								
N0	292 (98.3%)	399 (96.8%)	1.000	0.624	408 (98.3%)	283 (97.6%)	1.000	0.496
N1	5 (1.7%)	9 (2.2%)	1.317 (0.437–3.972)		7 (1.7%)	7 (2.4%)	1.442 (0.500–4.155)	
Clinical M stage								
M0	294 (99.0%)	400 (98.0%)	1.000	0.315	412 (99.3%)	282 (97.2%)	1.000	**0.032** *
M1	3 (1.0%)	8 (2.0%)	1.960 (0.516–7.451)		3 (0.7%)	8 (2.8%)	**3.896 (1.025–14.812)**	
Pathologic T stage								
2	159 (53.5%)	214 (52.5%)	1.000	0.776	224 (54.0%)	149 (51.4%)	1.000	0.497
3 + 4	138 (46.5%)	194 (47.5%)	1.044 (0.774–1.409)		191 (46.0%)	141 (48.6%)	1.110 (0.822–1.499)	
Pathologic N stage								
N0	278 (93.6%)	367 (90.3%)	1.000	0.086	389 (93.7%)	256 (88.3%)	1.000	**0.011** *
N1	19 (6.4%)	41 (9.7%)	1.635 (0.928–2.878)		26 (6.3%)	34 (11.7%)	**1.987 (1.164–3.391)**	
Seminal vesicle invasion								
No	235 (79.1%)	319 (78.2%)	1.000	0.764	328 (79.0%)	226 (77.9%)	1.000	0.725
Yes	62 (20.9%)	89 (21.8%)	1.057 (0.734–1.524)		87 (21.0%)	64 (22.1%)	1.068 (0.741–1.537)	
Perineural invasion								
No	81 (27.3%)	105 (25.7%)	1.000	0.647	112 (27.0%)	74 (25.5%)	1.000	0.663
Yes	216 (72.7%)	303 (74.3%)	1.082 (0.772–1.518)		303 (73.0%)	216 (74.5%)	1.079 (0.767–1.518)	
Lymphovascular invasion								
No	257 (86.5%)	336 (82.4%)	1.000	0.134	358 (86.3%)	235 (81.0%)	1.000	0.062
Yes	40 (13.5%)	72 (17.6%)	1.377 (0.905–2.094)		57 (13.7%)	55 (19.0%)	1.470 (0.980–2.205)	
D'Amico classification								
Low risk/intermediate risk	151 (50.8%)	199 (48.8%)	1.000	0.588	216 (52.0%)	134 (46.2%)	1.000	0.127
High risk	146 (49.2%)	209 (51.2%)	1.086 (0.805–1.465)		199 (48.0%)	156 (53.8%)	1.264 (0.936–1.707)	
Biochemical recurrence								
No	202 (68.0%)	279 (68.4%)	1.000	0.917	285 (68.7%)	196 (67.6%)	1.000	0.760
Yes	95 (32.0%)	129 (31.6%)	0.983 (0.713–1.355)		130 (31.3%)	94 (32.4%)	1.052 (0.762–1.450)	

*Note*: ORs with their 95% CIs were estimated by logistic regression models.

* *p* < 0.05 as statistically significant.

**TABLE 4:** Odds ratios (ORs) and 95% confidence intervals (CIs) of the clinical status and *PTX3* rs2305619 and rs1840680 genotypic frequencies in 705 patients with prostate cancer.

Variable	rs2305619				rs1840680			
	GG (*N* = 274)	GA + AA (*N* = 431)	OR (95% CI)	*p*‐value	GG (*N* = 295)	GA + AA (*N* = 410)	OR (95% CI)	*p*‐value
PSA at diagnosis (ng/mL)								
≤10	134 (48.9%)	201 (46.6%)	1.000	0.556	142 (48.1%)	193 (47.1%)	1.000	0.781
>10	140 (51.1%)	230 (53.4%)	1.095 (0.809–1.483)		153 (51.9%)	217 (52.9%)	1.044 (0.773–1.408)	
Pathologic Gleason grade group								
1 + 2 + 3	232 (84.7%)	353 (81.9%)	1.000	0.340	248 (84.1%)	337 (82.2%)	1.000	0.514
4 + 5	42 (15.3%)	78 (18.1%)	1.221 (0.810–1.839)		47 (15.9%)	73 (17.8%)	1.143 (0.765–1.708)	
Clinical T stage								
1 + 2	238 (86.9%)	369 (85.6%)	1.000	0.641	255 (86.4%)	352 (85.9%)	1.000	0.824
3 + 4	36 (13.1%)	62 (14.4%)	1.111 (0.714–1.728)		40 (13.6%)	58 (14.1%)	1.050 (0.681–1.621)	
Clinical N stage								
N0	270 (98.5%)	421 (97.7%)	1.000	0.425	290 (98.3%)	401 (97.8%)	1.000	0.639
N1	4 (1.5%)	10 (2.3%)	1.603 (0.498–5.164)		5 (1.7%)	9 (2.2%)	1.302 (0.432–3.925)	
Clinical M stage								
M0	271 (98.9%)	423 (98.1%)	1.000	0.427	292 (99.0%)	402 (98.0%)	1.000	0.323
M1	3 (1.1%)	8 (1.9%)	1.708 (0.449–6.496)		3 (1.0%)	8 (2.0%)	1.937 (0.510–7.364)	
Pathologic T stage								
2	148 (54.0%)	225 (52.2%)	1.000	0.639	159 (53.9%)	214 (52.2%)	1.000	0.655
3 + 4	126 (46.0%)	206 (47.8%)	1.075 (0.794–1.457)		136 (46.1%)	196 (47.8%)	1.071 (0.793–1.445)	
Pathologic N stage								
N0	256 (93.4%)	389 (90.3%)	1.000	0.141	276 (93.6%)	369 (90.0%)	1.000	0.095
N1	18 (6.6%)	42 (9.7%)	1.536 (0.865–2.727)		19 (6.4%)	41 (10.0%)	1.614 (0.917–2.842)	
Seminal vesicle invasion								
No	220 (80.3%)	334 (77.5%)	1.000	0.377	233 (79.0%)	321 (78.3%)	1.000	0.826
Yes	54 (19.7%)	97 (22.5%)	1.183 (0.814–1.719)		62 (21.0%)	89 (21.7%)	1.042 (0.723–1.502)	
Perineural invasion								
No	76 (27.7%)	110 (25.5%)	1.000	0.515	82 (27.8%)	104 (25.4%)	1.000	0.470
Yes	198 (72.3%)	321 (74.5%)	1.120 (0.796–1.577)		213 (72.2%)	306 (74.6%)	1.133 (0.808–1.589)	
Lymphovascular invasion								
No	236 (86.1%)	357 (82.8%)	1.000	0.243	256 (86.8%)	337 (82.2%)	1.000	0.100
Yes	38 (13.9%)	74 (17.2%)	1.287 (0.842–1.968)		39 (13.2%)	73 (17.8%)	1.422 (0.933–2.167)	
D'Amico classification								
Low risk/intermediate risk	140 (51.1%)	210 (48.7%)	1.000	0.539	151 (51.2%)	199 (48.5%)	1.000	0.488
High risk	134 (48.9%)	221 (51.3%)	1.110 (0.812–1.489)		144 (48.8%)	211 (51.5%)	1.112 (0.824–1.500)	
Biochemical recurrence								
No	185 (67.5%)	296 (68.7%)	1.000	0.747	201 (68.1%)	280 (68.3%)	1.000	0.965
Yes	89 (32.5%)	135 (31.3%)	0.948 (0.685–1.312)		94 (31.9%)	130 (31.7%)	0.993 (0.720–1.369)	

*Note*: ORs with their 95% CIs were estimated by logistic regression models.

### 3.3 Age‐specific effect of PTX3 rs3816527 on PCa progression

Since a genetic anchor of *PTX3* rs3816527 with the development of PCa was observed, we next explored whether there is any joint effect of rs3816527 and age on clinical characteristics of PCa. Intriguingly, our stratification analysis revealed that the association of rs3816527 with advanced forms of PCa (AC + CC: AA, pathologically‐confirmed nodal spread, OR, 3.241; 95% CI, 1.079–9.736; *p* = 0.028) (AC + CC: AA, metastatic tumour, OR, 9.589; 95% CI, 1.139–80.703; *p* = 0.012) was only detected in the younger age group (≤65 years old at cancer diagnosis) (Table 5). However, such correlation was not seen in the older age group (>65 years old at cancer diagnosis). These data indicate an age‐specific effect of *PTX3* rs3816527 on the progression of PCa.

**TABLE 5:** Odds ratios (ORs) and 95% confidence intervals (CIs) of the clinical status and *PTX3* rs3816527 genotypic frequencies in 705 patients with prostate cancer with different age at diagnosis.

Variable	Age at diagnosis ≤65 (*N* = 298)				Age at diagnosis >65 (*N* = 407)			
	AA (*N* = 180)	AC + CC (*N* = 118)	OR (95% CI)	*p*‐value	AA (*N* = 235)	AC + CC (*N* = 172)	OR (95% CI)	*p*‐value
PSA at diagnosis (ng/mL)								
≤10	100 (55.6%)	61 (51.7%)	1.000	0.513	106 (45.1%)	68 (39.5%)	1.000	0.262
>10	80 (44.4%)	57 (48.3%)	1.168 (0.733–1.861)		129 (54.9%)	104 (60.5%)	1.257 (0.843–1.874)	
Pathologic Gleason grade group								
1 + 2 + 3	164 (91.1%)	100 (84.7%)	1.000	0.091	185 (78.7%)	136 (79.1%)	1.000	0.933
4 + 5	16 (8.9%)	18 (15.3%)	1.845 (0.900–3.782)		50 (21.3%)	36 (20.9%)	0.979 (0.605–1.586)	
Clinical T stage								
1 + 2	169 (93.9%)	103 (87.3%)	1.000	0.059	198 (84.3%)	137 (79.7%)	1.000	0.229
3 + 4	11 (6.1%)	15 (12.7%)	2.237 (0.990–5.058)		37 (15.7%)	35 (20.3%)	1.367 (0.820–2.279)	
Clinical N stage								
N0	178 (98.9%)	115 (97.5%)	1.000	0.347	230 (97.9%)	168 (97.7%)	1.000	0.893
N1	2 (1.1%)	3 (2.5%)	2.322 (0.382–14.109)		5 (2.1%)	4 (2.3%)	1.095 (0.290–4.140)	
Clinical M stage								
M0	179 (99.4%)	112 (94.9%)	1.000	**0.012** *	233 (99.1%)	170 (98.8%)	1.000	0.753
M1	1 (0.6%)	6 (5.1%)	**9.589 (1.139–80.703)**		2 (0.9%)	2 (1.2%)	1.371 (0.191–9.827)	
Pathologic T stage								
2	102 (56.7%)	69 (58.5%)	1.000	0.758	122 (51.9%)	80 (46.5%)	1.000	0.281
3 + 4	78 (43.3%)	49 (41.5%)	0.929 (0.580–1.486)		113 (48.1%)	92 (53.5%)	1.242 (0.837–1.841)	
Pathologic N stage								
N0	175 (97.2%)	108 (91.5%)	1.000	**0.028** *	214 (91.1%)	148 (86.0%)	1.000	0.111
N1	5 (2.8%)	10 (8.5%)	**3.241 (1.079–9.736)**		21 (8.9%)	24 (14.0%)	1.653 (0.887–3.078)	
Seminal vesicle invasion								
No	149 (82.8%)	96 (81.4%)	1.000	0.754	179 (76.2%)	130 (75.6%)	1.000	0.891
Yes	31 (17.2%)	22 (18.6%)	1.101 (0.602–2.014)		56 (23.8%)	42 (24.4%)	1.033 (0.652–1.635)	
Perineural invasion								
No	49 (27.2%)	39 (33.1%)	1.000	0.281	63 (26.8%)	35 (20.3%)	1.000	0.132
Yes	131 (72.8%)	79 (66.9%)	0.758 (0.457–1.255)		172 (73.2%)	137 (79.7%)	1.434 (0.896–2.294)	
Lymphovascular invasion								
No	161 (89.4%)	96 (81.4%)	1.000	**0.047** *	197 (83.8%)	139 (80.8%)	1.000	0.428
Yes	19 (10.6%)	22 (18.6%)	**1.942 (1.000–3.772)**		38 (16.2%)	33 (19.2%)	1.231 (0.736–2.059)	
D'Amico classification								
Low risk/intermediate risk	111 (61.7%)	65 (55.1%)	1.000	0.258	105 (44.7%)	69 (40.1%)	1.000	0.358
High risk	69 (38.3%)	53 (44.9%)	1.312 (0.819–2.101)		130 (55.3%)	103 (59.9%)	1.206 (0.809–1.797)	
Biochemical recurrence								
No	128 (71.1%)	79 (66.9%)	1.000	0.446	157 (66.8%)	117 (68.0%)	1.000	0.796
Yes	52 (28.9%)	39 (33.1%)	1.215 (0.736–2.006)		78 (33.2%)	55 (32.0%)	0.946 (0.622–1.440)	

*Note*: ORs with their 95% CIs were estimated by logistic regression models.

* *p* < 0.05 as statistically significant values in bold.

### 3.4 Insight of rs3816527 variant into PTX3 protein structure and oligomerization

**FIGURE 1:**
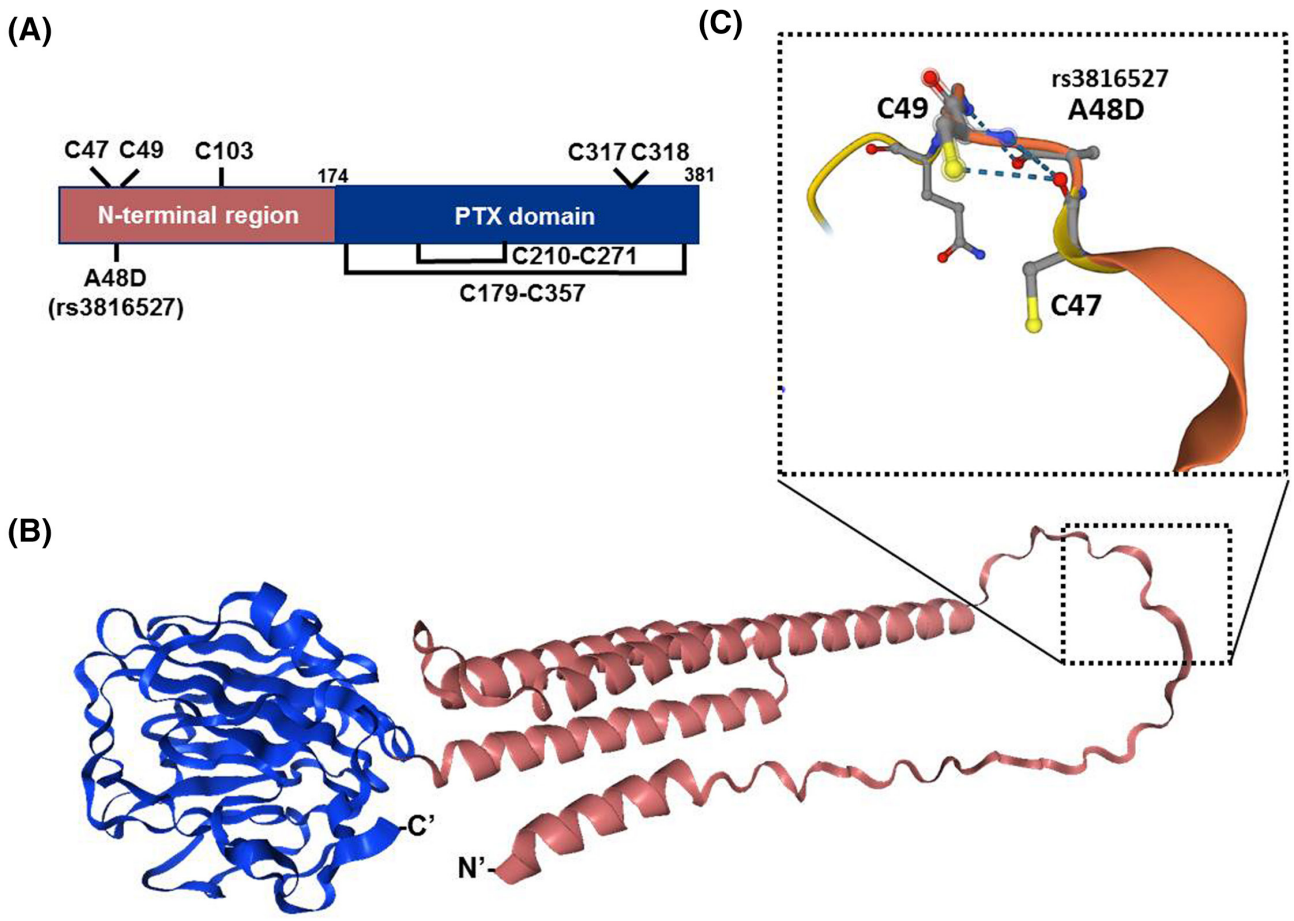
Conformational insights into the human variant (rs3816527) revealed the formation of the quaternary organization through altering orders of PTX3 protein structure. (A) Schematic representing the protein domains of PTX3 monomer (UniProt: P26022), highlighting the locations of cysteine bond networks. The closed loops illustrate the presence of intra‐monomeric disulfide bonds. C47/C49 could participate in intra‐ or inter‐subunit disulfide bonds, and C317/C318 in inter‐tetramer disulfide bonds to form PTX3 oligomerization states. Domain symbols are drawn approximately to scale. The numbering of residues is displayed in colour black. (B) Ribbon diagram (NGL viewer 2.0) for the 3D AlphaFold‐based predicted model of human PTX3 (https://alphafold.ebi.ac.uk/entry/P26022) includes the C‐terminal PTX domain core (Pfam: PF00354; blue) and the N‐terminal tetrameric coiled‐coil domain (red) that is connected by conserved cysteine sulfur bonds. (C) Enlarged view of the selected variant region, displayed in a similar orientation as shown in part (B). The representation used a ball‐and‐stick model and broken lines to mark the hydrogen‐bonding interactions between backbone groups.

To gain additional insight of rs3816527 in PTX3 function, structural information of PTX3 protein was predicted (Figure 1). The amino acid residue (A48D) encoded by rs3816527 allele of *PTX3* gene is located between two cysteines, C47 and C49. It has been reported that these two cysteine residues exert a vital role in PTX3 covalent oligomerization by establishing inter‐chain symmetric disulfide bridges (C47‐C47 and C49‐C49).^33^ Our structural prediction revealed that the presence of polymorphic rs3816527 allele likely facilitates the formation of intra‐chain asymmetric disulfide bonds (C47‐C49), thus altering the quaternary organization and oligomerization of PTX3 protein.

### 3.5 Induction of PTX3 in PCa is required for cell migration and associated with tumour metastasis

**FIGURE 2:**
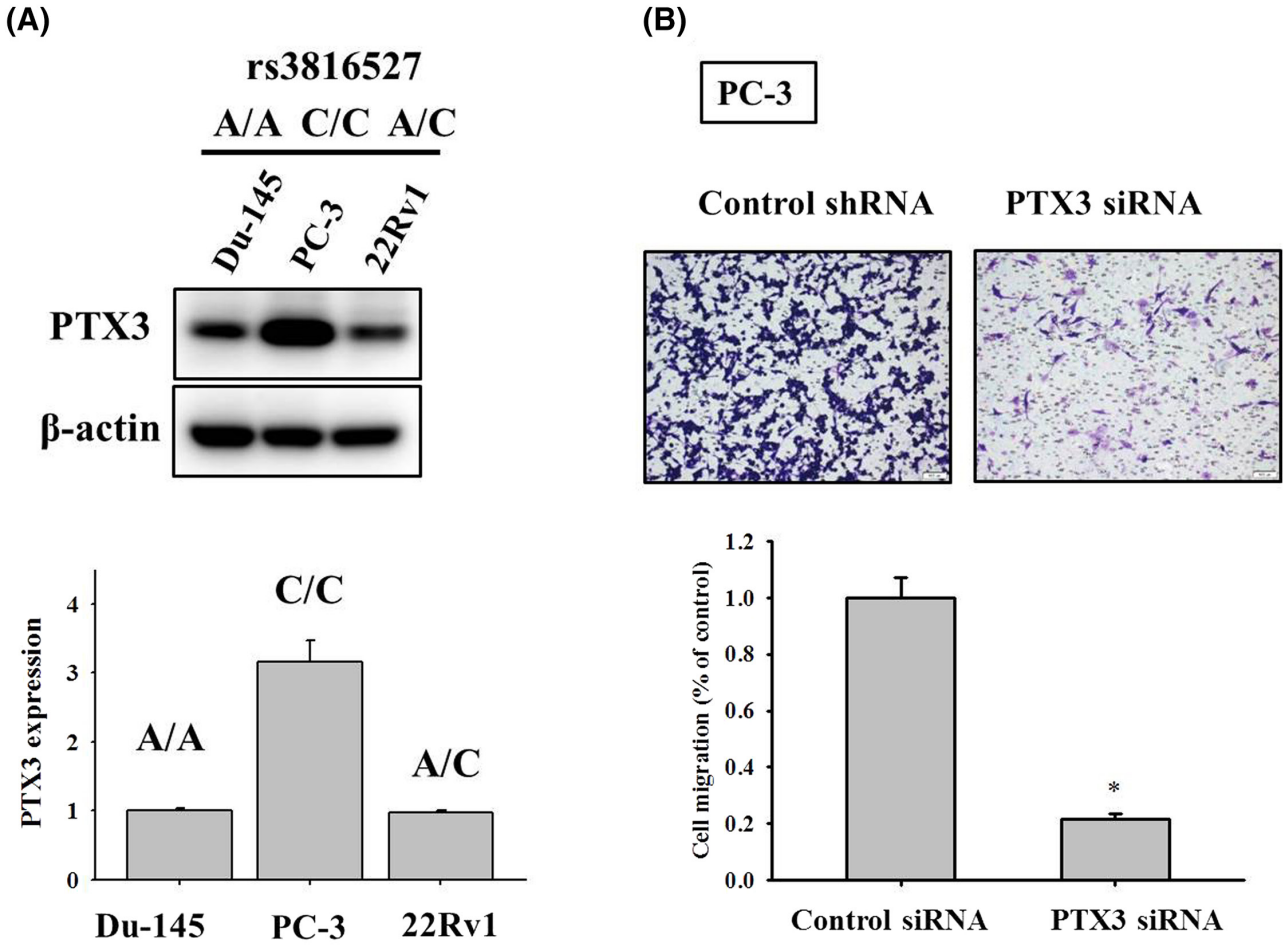
Silencing of *PTX3* gene impaired cell migration and clonogenic survival of PCa cells. (A) Basal levels of PTX3 in three PCa cell lines, whose rs3816527 genotypes were determined, were evaluated via immunoblotting. (B) PC‐3 cells transfected with vectors expressing control or PTX3‐specific siRNA were seeded in a modified Boyden chamber for measurement of cell migration. Quantitative data are shown underneath. *p < 0.05 as compared with scramble shRNA controls.

**FIGURE 3:**
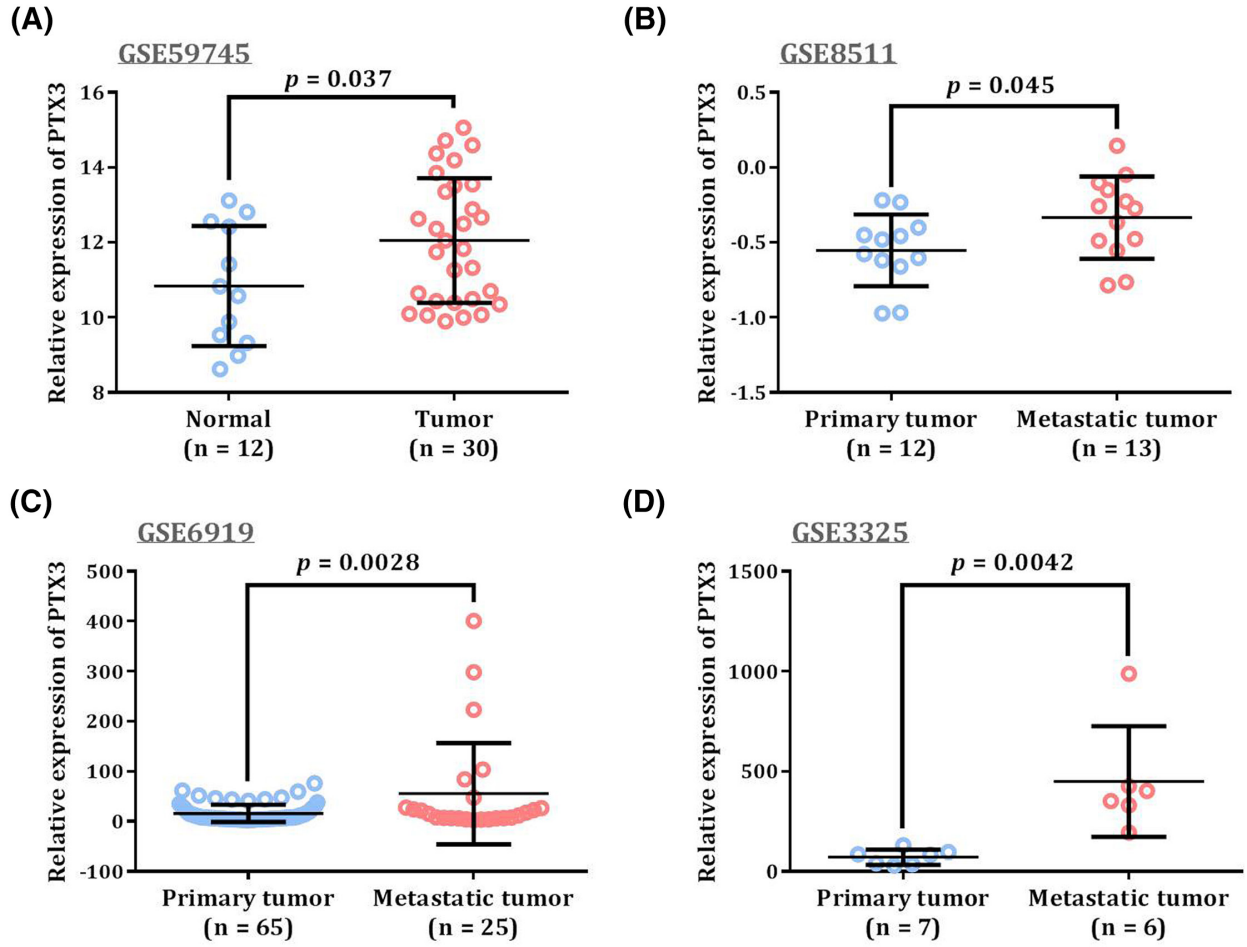
Comparison of PTX3 levels in PCa datasets from the Gene Expression Omnibus (*GEO*) repository. (A) PTX3 levels between PCa samples and adjacent normal prostate tissues in GSE59745. (B‐D) PTX3 levels between primary and metastatic PCa specimens in GSE8511 (B), GSE6919 (C), and GSE3325 (D). *p*‐values were calculated between two groups by Student's *t*‐test.

We next evaluated PTX3 expression in three androgen‐independent PCa cell lines. Differential expression of PTX3 was observed in cell lines possessing different rs3816527 genotypes, of which PC‐3 cells (homozygous for the minor allele of rs3816527, CC) expressed a substantial level of PTX3 compared to that of Du‐145 (carrying homozygous A allele of rs3816527) and 22Rv1 cells (carrying both A and C allele of rs3816527) (Figure 2A). Silencing of *PTX3* gene in PC‐3 cells significantly impaired the potential of cell migration (Figure 2B), suggesting a functional role of PTX3 in PCa cell migration. Moreover, to explore clinical relevance of PTX3 levels in PCa progression, PTX3 expression in several datasets of the Gene Expression Omnibus (*GEO*) repository was analysed. We found that PTX3 levels of PCa tumour samples were higher than that of normal adjacent prostate tissues in GSE59745 (Figure 3A). In additional three datasets collected for studying PCa metastasis, an elevation of PTX3 expression was consistently detected in metastatic tumours as compared to primary cancer samples (Figure 3B–D). These data collectively support a link of PTX3 induction to cell migration and metastatic response of PCa.

## 4 DISCUSSION

It is well recognized that the development and progression of prostate tumorigenesis are under the influence of many genetic components that confer different levels of cancer risk. Here, a correlation between *PTX3* rs3816527 variations and an increased propensity for the development of advanced forms of disease was demonstrated in patients with PCa. Surprisingly, such association of rs3816527 with PCa progression was only seen among younger patients, suggesting an impact of age on genetic risks for PCa. Differential expression of PTX3 was observed in PCa cell lines that harbour different rs3816527 genotypes. Our in vitro experiments and survey of publicly available PCa datasets exhibited that elevated expression of PTX3 in PCa was required for cell migration and associated with tumour metastasis. The previous study also revealed that PTX3 promotes breast cancer cell proliferation and metastasis.^34^ Collectively, our results revealed a link of *PTX3* rs3816527 to the progression of PCa.

Polymorphism (A/C) of rs3816527 in humans gives rise to an amino acid replacement with an alanine residue located at an evolutionarily conserved region of PTX3 protein across the lineage of mammals, all of which, instead, carry an aspartic acid at this position (position 48).^35^ *PTX3* rs3816527 genotypes have been shown to confer the predisposition to numerous conditions, including microbial infection,^35^, ^36^, ^37^, ^38^ hypertension,^39^ migraine,^40^ and oral malignancy.^25^ In an in silico analysis of functional *PTX3* SNPs in the defence against infectious pathogens,^41^ rs3816527 was predicted as a deleterious non‐synonymous SNP via the SNAP algorithm.^42^ This amino acid substitution (D48A) was proposed to damage the electrostatic potential of PTX3 protein structure, thereby affecting interactions with other proteins.^35^ In addition, two adjacent positions of PTX3 D48A encode cysteine residues, C47 and C49, which have been shown to establish inter‐subunit symmetric disulfide bridges (C47‐C47 and C49‐C49) in the formation of PTX3 oligomer.^33^ However, C47 and C49 of PTX3 can also participate in inter‐subunit asymmetric disulfide bonds (C47‐C49).^43^ Our prediction of PTX3 protein structure further supported this notion and revealed a change in the quaternary organization and oligomerization of PTX3 protein due to the disordered nature of this area partly attributed by the presence of polymorphic rs3816527 allele.

In addition to altered protein conformation and ligand binding, expression levels of PTX3 may be also regulated due to polymorphic alleles. It has been reported that the nucleotide substitution (A/C) of rs3816527 severely affected the folding of PTX3 mRNA, indicating a change in mRNA stability.^35^ In the same study, lower levels of PTX3 mRNA and protein were detected in promyelocytes carrying homozygous A allele of rs3816527, as compared to those carrying homozygous C allele. Similar genotypic effects on basal levels of PTX3 were also observed among three PCa cell lines examined in our study. Even though the C allele of rs3816527 was also predicted to potentially create a cryptic splicing site for generation of a smaller transcript, alternative splicing is nevertheless unable to be verified.^36^ Taken together, our results and findings from others suggest that elevated levels of PTX3 by virtue of altered mRNA secondary structure attributed by rs3816527 genotypes promote cell migration and metastatic potential in PCa.

One intriguing finding of this study is that the correlation of rs3816527 with PCa progression was only detected among younger patients, indicating an impact of age on genetic risks for PCa. It has been noted that for some diseases, genetic risk parameters exhibit stronger explanatory power among younger populations, compared to older ones.^44^ Such tendency for genetic risks to decline with increasing age was previously documented in prostate malignancy^45^ and other conditions.^46^, ^47^, ^48^ However, genetic risk factors are not equally relevant to human disorders across age contexts, though the reasons for such variation are not clear. By applying a proportional hazards model within an interval‐based censoring methodology to data from the UK Biobank, several aspects of the relationship between age and genetic relative risk have been proposed.^44^ First, for some but not all diseases, a non‐constant correlation between age and the influence of genetic risk was statistically verified. In such cases, genetic risks conferred the largest impact at earlier ages, although the trend and magnitude of the drop‐off varied among diseases. Moreover, the drop‐off in genetic association with age cannot be ascribed to hidden variation in unmeasured covariates such as environmental factors. These perspectives support our observation that PCa is one of such diseases affected by age‐varying genetic risk profiles.

This study unveiled an age‐specific effect of PTX3 gene polymorphisms on lymphatic and distal metastasis of PCa. Nevertheless, additional efforts are needed to deal with several limitations of the current work. One is that the mechanism underlying the regulation of *PTX3* rs3816527 in PCa progression and spread remains elusive, as serum levels of PTX3 have proved a better performance in identifying PCa patients than the serum PSA curve.^49^ More explorations are required to clarify whether *PTX3* rs3816527 modulates its own expression at the transcriptional or post‐transcriptional level, and further PTX3 protein crystallization may help to determine whether rs3816527 polymorphisms alter the formation of PTX3 oligomers or perturb its affinity to binding partners. Another concern is that selection bias may arise in age‐stratified analyses. Conducting a longitudinal analysis of PCa risk could be an alternative experimental design to address the impact of age on the association of *PTX3* rs3816527 with PCa. In addition, our findings might be merely applicable to unique ethnic populations and require extra replication cohorts to verify the association.

In conclusion, our analysis showed a relationship between *PTX3* rs3816527 and the risk for the development of advanced PCa. This genetic susceptibility was only detected among younger cases but not for older ones, indicating an age‐dependent effect of *PTX3* polymorphisms on the progression of PCa. Furthermore, increased expression of PTX3 in PCa was found to be indispensable for cancer cell migration and correlated with tumour metastasis. These data link *PTX3* gene polymorphisms to the metastatic potential of PCa.

## AUTHOR CONTRIBUTIONS

**Wei‐Chun Weng:** Conceptualization (equal); data curation (equal); writing – original draft (equal); writing – review and editing (equal). **Yi‐Hsien Hsieh:** Conceptualization (equal); methodology (equal). **Chia‐Yen Lin:** Data curation (equal); resources (equal). **Yu‐Fan Liu:** Data curation (equal); software (equal). **Shih‐Chi Su:** Data curation (equal); methodology (equal); writing – original draft (equal); writing – original draft (equal). **Shian‐Shiang Wang:** Conceptualization (equal); data curation (equal); resources (equal); writing – review and editing (equal). **Shun‐Fa Yang:** Conceptualization (equal); data curation (equal); writing – original draft (equal); writing – review and editing (equal).

## CONFLICT OF INTEREST STATEMENT

The authors declare no conflicts of interest related to this study.

## Data Availability

The data used to support the findings of this study are available from the corresponding author upon reasonable request.
